# Posterior only instrumented fusion provides incomplete curve control for early-onset scoliosis in type 1 neurofibromatosis

**DOI:** 10.1186/s12887-020-1956-y

**Published:** 2020-02-10

**Authors:** Siyi Cai, Zhengyao Li, Guixing Qiu, Jianxiong Shen, Hong Zhao, Yu Zhao, Yipeng Wang, Jianguo Zhang

**Affiliations:** Department of Orthopedics, Peking Union Medical College Hospital, 1 Shuaifuyuan Rd, Peking, 100730 China

**Keywords:** Early-onset scoliosis, Neurofibromatosis, Posterior fusion, Surgical outcome, Long-term

## Abstract

**Background:**

The mid-long term outcomes of posterior spinal fusion in pediatric neurofibromatosis type 1 (NF-1) patients are rarely reported, so does the effectiveness of itsorthopeidc maintenance function. This study aims to evaluate the mid-long term surgical outcomes of posterior only instrumented spinal fusion for early-onset scoliosis (EOS) in NF-1 patients.

**Methods:**

A retrospective review was performed on a cohort of 10 NF-1 patients having EOS from 2008 to 2014 in our hospital, the age averaged at 7.8 years old when they underwent posterior only instrumented spinal fusion for their EOS. Both general clinical data and surgical specific data of the patients were collected and reviewed, and the dystrophic progression of EOS was evaluated during the follow-up.

**Results:**

The average duration of follow-up was 54 months (24 to 88 months). All patients underwent posterior only instrumented spinal fusion at 1 stage. The primary curves of EOS were thoracic in 9 cases and 1 patient had lumbar scoliosis. Preoperative major curve was significantly corrected (from 66.1 to 31.1 degrees). However, the major curve deteriorated significantly to 40.1 degrees on average at the end of the follow-up. The T1-S1 distance increased 2.8 cm on average and kept increasing at a rate of 0.6 cm/year during the follow-up.

**Conclusions:**

Posterior only fusion surgery was not a good option to treat the EOS in NF-1 patients despite the relatively short segments involvement in the disease. The maintenance of orthopedic effect after treatment was not satisfactory.

## Background

Neurofibromatosis type 1 (NF-1) is an uncommon neurocutaneous disorder caused by autosomal dominant mutations on chromosome 17q11.2. It was first described by Von Recklinghausen in 1882 with typical manifestations of various deteriorations of skin, bones, arteries, peripheral nerves and central nervous system. The prevalence of NF-1 is from 1/4000 to 1/5000 [1]. Ten to 60 % of the NF-1 patients demonstrate the symptom of early onset spinal deformity, either dystrophic or non-dystrophic [2]. Typical NF-1 dystrophic scoliosis shows a short, sharp curve and can be diagnosed by having 3 or more characteristic dystrophic features such as rib penciling, vertebral scalloping, wedging, rotation and spindling of the transverse process. Given that the dystrophic scoliosis in NF-1 patients has a tendency of curve progression which usually results in severely poor pulmonary function and trunk height loss, aggressive treatments are recommended to treat the disease [3–5]. While about 50% of the performed fusion surgeries involve the fusion of additional segments, application of the growing rod during the fusion suggests longer segments of the spinal [6] However, due to the short and sharp curve of the dystrophic scoliosis in NF-1 patients, some surgeons suggested that the short segments fusion technique should be used to correct the spinal deformity in NF-1 patients to provide better postoperative motor function and eventually, better ability to do common activities in daily life.

Anterior-posterior fusion has been widely used to treat dystrophic scoliosis in NF-1 patients [7, 8]. . Recently, the posterior only instrument fusion was reported to be used to treat dystrophic scoliosis in NF-1 patients because challenges for the anterior approach caused by the extensive plexiform tumors could be avoided by using posterior approach and a good short-term result from posterior approach has been reported [9, 10]. In Li and his colleagues’ study, the posterior only approach demonstrated a good short-term result to treat early-onset scoliosis (EOS) in a group of NF-1 patients with an average age of 13 (the youngest patient was 8 years old), but the number of included patients were not addressed [10]. However, it is rarely reported whether posterior spinal fusion will achieve a good mid-long-term result in patients at young age (age < 10Yrs) [11].

The aim of this study is to evaluate the mid-long-term surgical outcomes of posterior fusion instrument only surgeries for dystrophic EOS in NF-1 patients.

## Methods

### Patients

guardians was obtained. And written informed consent was obtained from the patient/parents/legal guardians for publication of any accompanying images and videos. A copy of the written consent is available for review by the editor of this journal. Each included patient was diagnosed by using the established diagnostic criteria [8, 12]. All the included patients met the diagnosis criteria of dystrophic EOS. A total number of 94 patients received surgeries from March 2008 to March 2014 in our hospital, twenty-six of them with dystrophic EOS underwent the initial surgery at an age younger than 10.

Sixteen cases of the 26 patients mentioned above were further excluded from our study including 3 patients who underwent anterior-posterior fusions, 2 patients who had one stage posterior osteotomy with short segment fusions, 8 patients who received growing rod instrumentation and 3 patients whose follow-up duration was less than 2 years. Finally, 10 NF-1 patients with dystrophic EOS (aged at 7.8 ± 2.1 years) were included in this study.

### Data collection

General clinical data including initial surgery age, body mass index (BMI), American Spinal Injury Association (ASIA) score and follow-up duration were collected. Collected surgery related data included the number of involved surgical segments, the type of anchor instrumentation (hook, screw or hybrid), instrumentation intensity, diameters of the rod, bone grafting strategy (material and location), intraoperative neurophysiology monitor, operation time, blood loose and blood transfusion.

### Radiographic features

The collected radiographic information included the major coronal curve, the sagittal curve of T2-T5, T5-T12 and T10-L2, the lengths of T1-S1 and T1-T12 and space available for the lung salmeterol (SAL). The proximal and distal junctional kyphosis were measured at the time of post-operation and last follow-up. Eight cases have been found dural ectasia on preoperative Magnetic resonance (MR) image and 2 cases had paraspinal tumors or plexiform neurofibromas located close to the scoliotic curve. The dystrophic extent were reevaluated at the last follow-up through radiographing.

### Pulmonary function

Pulmonary function tests were performed by well-trained technician prior to the surgery and each follow-up. All patients underwent pulmonary function tests performed on PFT (Cardinal healthcare Germany), the Forced vital capacity (FVC), Forced Expiratory Volume in the first second (FEV1), and FEV1/FVC were measured by spirometry, which was performed according to the American Thoracic Society (ATS) standards.

### Surgical procedures

The preoperative traction was not used. All patients were preferred to be treated with screw-based instrumentation. In order to minimize the fusion segments, we refer to the fusion range of adolescent idiopathic scoliosis. The range of spinal fusion is from the upper end vertebra to the lower stable vertebra. If the pedicle was not large enough or failed in fixation, hooks were used in addition to screws. Correction of deformity was achieved by a combination of rod derotation and sequential in situ translational reduction, with or without in situ bending of the rod.

The curve contained 4.8 segments on average (4 to 5); the average number of fusion segment was 8 (4 to 13). Implant density was defined as the ratio between the total number of anchor points of the internal fixation to the number of fusion segments. In our study, the average implant density was 1.2 (0.9 to 1.6). Three patients were operated with both pedicle screws and hooks, among them two patients were operated with two hooks and the third patient had one hook implanted. Five patients used the connectors including four patients having one connector and the fifth patient having two connectors. All patients used titanium rods. The diameter of the rods used was 5.5 mm except for two patients whose rod diameter was 4.5 mm.

Additional correction maneuvers, including appropriate compression and distraction, were performed to provide 3D correction of the deformity. The posterior elements were decorticated, and bone grafts were placed on the decorticated bed using autogenous local bone grafts in combination with allogeneic bone grafts.

The average blood loss during the surgery was 580 ml (200-2000 ml). Three patients were given blood transfusions with an average volume of 506 ml. Two of three patients used cell saver to save the blood (300 ml, 2000 ml, respectively), and 120 ml and 1000 ml of the saved blood was transfused back. The two patients who had the most blood loss received the concave side para-spinal tumor resection during the procedure. Hard braces were used after surgeries for 6–8 months in all patients.

### Statistical analysis

Statistical analyses were performed with SPSS (IBM, New York, USA) software, version 22.0 for Windows. Independent sample t-test was performed when comparing two groups; if the variances were not equal, Wilcoxon test was applied. One-Way ANOVA was used to compare the means among three groups.

Results were presented as mean ± SD, unless otherwise indicated. The differences were considered significant if *P* < 0.05; *P* values between 0.05 and 0.10 were considered as the difference in trends.

## Results

### Significant coronal curve correction but poor maintenance

Ten NF-1 patients with dystrophic scoliosis were included in this study. Detailed characteristics of each patient were described in Table 1. The average age of patients at the initial surgery was 7.8 years (4.2 to 9.6 years). The average follows-up duration was 54 months (24 to 88 months). There were 9 cases of thoracic scoliosis and one case of lumbar scoliosis.

**Table 1:** Clinical and radiographic data of 10 NF-1 patients with early-onset Scoliosis treated by posterior only fusion

No	Age	FU (Mon)	MC	Pre-O MC (cobb)°	Post-O MC (cobb)°(%)	FU MC (cobb)°(%)	Pre-O T5–12 kyphosis (cobb)°	Post-O T5–12 kyphosis (cobb)°(%)	FU T5–12 kyphosis (cobb)°(%)	Pre-O T12-S1 Lordosis (cobb)°	Post-O T12-S1 Lordosis (cobb)°(%)	FU T12-L1 Lordosis (cobb)°(%)	Pre-O T10-L2 Lordosis (cobb)°	Post-O T10-L2 Lordosis (cobb)°(%)	FU T10-L2 Lordosis (cobb)°(%)	Pre-O T1S1(cm)	Post-O T1S1 (cm)	FU T1S1 (cm)	T1S1 gain/year (cm)	Pre-O T1–12(cm)	Post-O T1–12(cm)	FU T1–12(cm)	T1–12 gain/year (cm)
1	9.1	24	L1–4	51.5	25 (51%)	41.2 (20%)	16.5	41.8(− 153%)	39.4(− 139%)	58.0	66.6(−15%)	59.8(−3%)	− 5.0	5.8 (216%)	5 (200%)	33.6	35.1	37.9	1.4	22.1	22.2	25.4	1.6
2	7.2	61	T10-L2	70.0	21 (70%)	39 (44%)	22.9	27.6(−21%)	14.2 (38%)	52.7	45.8 (13%)	40.6 (23%)	2.9	1.3 (55)	12.5(− 331%)	30.2	31.8	34.1	0.4	19.6	20.3	22.1	0.4
3	8.3	88	T5–9	88.0	55 (38%)	70.3 (20%)	47.7	32.3 (32%)	38.5 (19%)	49.9	17.5 (65%)	43.1 (14%)	9.2	15.3(−66%)	1.9 (79%)	36.1	39.5	43.2	0.5	22.1	24.7	25.6	0.1
4	4.2	35	T6–9	43.0	27.1 (37%)	27.4 (36%)	60.8	24.8 (59%)	34.9 (43%)	48.4	35.5 (27%)	58.2(−20%)	24.1	7.5 (69%)	10.9 (55%)	38.7	41.9	43.9	0.7	24.9	26.9	27.8	0.3
5	7.1	49	T5–9	83.3	45.3 (46%)	57.7 (31%)	70.5	35 (50%)	62.7 (11%)	66.0	22.6 (66%)	66.2 (0%)	3.1	12.7(−310%)	4.2(−35%)	36.0	39.5	41.9	0.6	20.4	23.8	24.4	0.1
6	4.1	34	T7–11	60.9	30.8 (49%)	41.4 (32%)	26.0	20 (23%)	30.9(−19%)	47.6	46.8 (2%)	54.2(−14%)	1.0	1.5(−50%)	6.5(− 550%)	26.7	29.0	31.4	0.8	16.7	18.4	19.7	0.5
7	9.2	67	T6–10	59.6	13.4 (78%)	27.2 (54)		6.2	20.6	52.5	44.7 (15%)	62.7(−19%)	23.0	5.3 (77%)	8 (65%)	42.5	44.6	46.7	0.4	26.8	27.4	29.6	0.4
8	9.5	68	T5–8	90.3	51.2 (43%)	54 (40%)	56.8	46.3 (18%)	50.7 (11%)	46.4	43.5 (6%)	45.6 (2%)	8.0	6.5 (19%)	7.4 (7.5%)	30.8	34.3	36.4	0.4	16.7	20.4	20.6	0.0
9	9.6	62	T5–9	58.9	27.4 (53%)	34.3 (42%)	66.5	26.8 (60%)	34.6 (48%)	65.7	39 (41%)	43 (35%)	6.7	2 (70%)	18.4(− 175%)	32.4	37.0	39.4	0.5	20.5	23.4	24.0	0.1
10	9.6	52	T8–12	55.0	15 (73%)	17 (69%)	18.0	20(−11%)	19(−1%)	30.5	43.8(−44%)	44.7(− 47%)	9.2	0.9 (90%)	4.9 (47%)	38.0	40.0	43.2	0.7	24.0	25.1	25.6	0.1

*PF* Posterior only fusion, *GR* Traditional growing rod, *FU* Follow-up, *Pre-O* Preoperative, *Post-O* Postoperative, *MC* Major curve

The major curve was significantly corrected from 66.1° ±16.2° (43.0 to 90.3 degrees) to 31.1° ±14.6° (13.4 to 51.2 degrees) after the initial surgery, the coronal curve correction rate was 54% ± 14%, (*P* = 0.00). The average major curve at the last follow-up increased significantly to 41.0 ° ±16.0 ° (17.0 to 70.3 degrees) compared to the initial result (*P* = 0.001). The coronal curve correction rate was 39 ± 15% at the last follow-up. The difference was statistically significant (P = 0.001). The mean rotation of apex vertebrate was corrected from 2 (1 to 3) preoperatively to 1.8 (1 to 3) after initial surgery and was maintained at 1.9 (1 to 3) at the last follow-up. In one case, the preoperative T5-T12 kyphosis angle was not recognizable because of the low quality of the film. Analysis of the data from the other 9 patients demonstrated that the T5-T12 kyphosis angle was corrected from 42.9° ±22.0°, preoperatively, to 28.1° ± 11.6°, post initial-operatively, without statistically significant change (*P* = 0.08). This was also true for the change of T5-T12 kyphosis angle from the initial post operation to the last follow up (follow-up T5–12, 34.6° ± 14.7°, *P* = 0.09). Similarly, with regards to the T1-S1 kyphosis angle, there was no significant difference was found between preoperatively and post initial-operatively, or between post initial-operatively and the last follow up.

### Continued spinal growth during the growth phase

The T1-S1 length was 34.5 cm ±4.7 cm before operation and 39.8 cm ±4.8 cm, with an average increase of 15.6% ± 3.6% (5.3 cm ±1.1 cm). During the follow-up, the T1-S1 length increased at the rate of 0.6 cm ±0.3 cm per year. The average T1-T12 distance increase ratio was 9.4% ± 6.7%, which increased from 21.4 cm ±3.3 cm to 23.3 cm ±3.0 cm after operation, and the lengthening velocity was 0.4 cm ±0.5 cm per year. The T1–12 growth velocity was significantly inhibited by the fusion operation compared to that of the T1-S1 (*P* = 0.007). SAL changed from 0.987 (0.854 to 1.145) to 1.008 (0.932 to 1.16) and was maintained at 1.035 (0.961 to 1.16).

### No neurological complication but high alignment complication

Only 1 case had perioperative complications on records (surgery wound superficial infection). One case had transient ileus; another case had transient urinary tract infection (reported but may not the true complications of the related surgery) (Table 2).

**Table 2:** Clinical data and surgical information on 10 NF-1 Patients with Early-onset Scoliosis Treated by posterior only fusion

No	Fusion or involved level	Anchor sites (Hooks)	Anchor sites nearby the apex level (upper 1/lower 1)	Rod diameter (mm)	Intraoperative blood lost (mm)	Transconnector (numbers)	Complications		
							Perioperative complication	Alighment complication	Implant related
1	T11-L5	6 (2)	N	5.5	500	1		the curve deteriorated to 37.6°at 2 years FU and worsen continuously	
2	T9-L3	11	Y	5	400	0		the curve deteriorated to 55°at 5 years FU and the thoracilumbar kyphosis deteriorated from 7.8°to 27°。	1 distal screw slipped at 5 years FU
3	T3–12	10 (4)	N	5.5	500	1	urinal infection	CP, MC increased 15.3°	
4	T6–9	4	N	4.5	400	1		decompensated lumbar curve.	1 screw dislodgement
5	T4–11	8	N	5.5	400	1	intestinal paralysis	CP, MC increased 13.4°	2 screws pulled out
6	T7–11	9	Y	4.5	400	0		CP, MC increased 11.4°, decompensated lumbar curve.	
7	T5–11	8	N	5.5	300	0		CP, MC increased 13.8°, decompensated lumbar curve.	
8	T2-L1	12 (2)	N	5.5	3000	2			
9	T2-L2	15	N	5.5	700	0			
10	T6-L1	11	Y	5.5	400	0	superficial infection		

*CP* Crankshaft phenomenon, *MC* Major Curve, *Postop* Postoperative, *PI* Post the Initial surgery

**Fig. 1:**
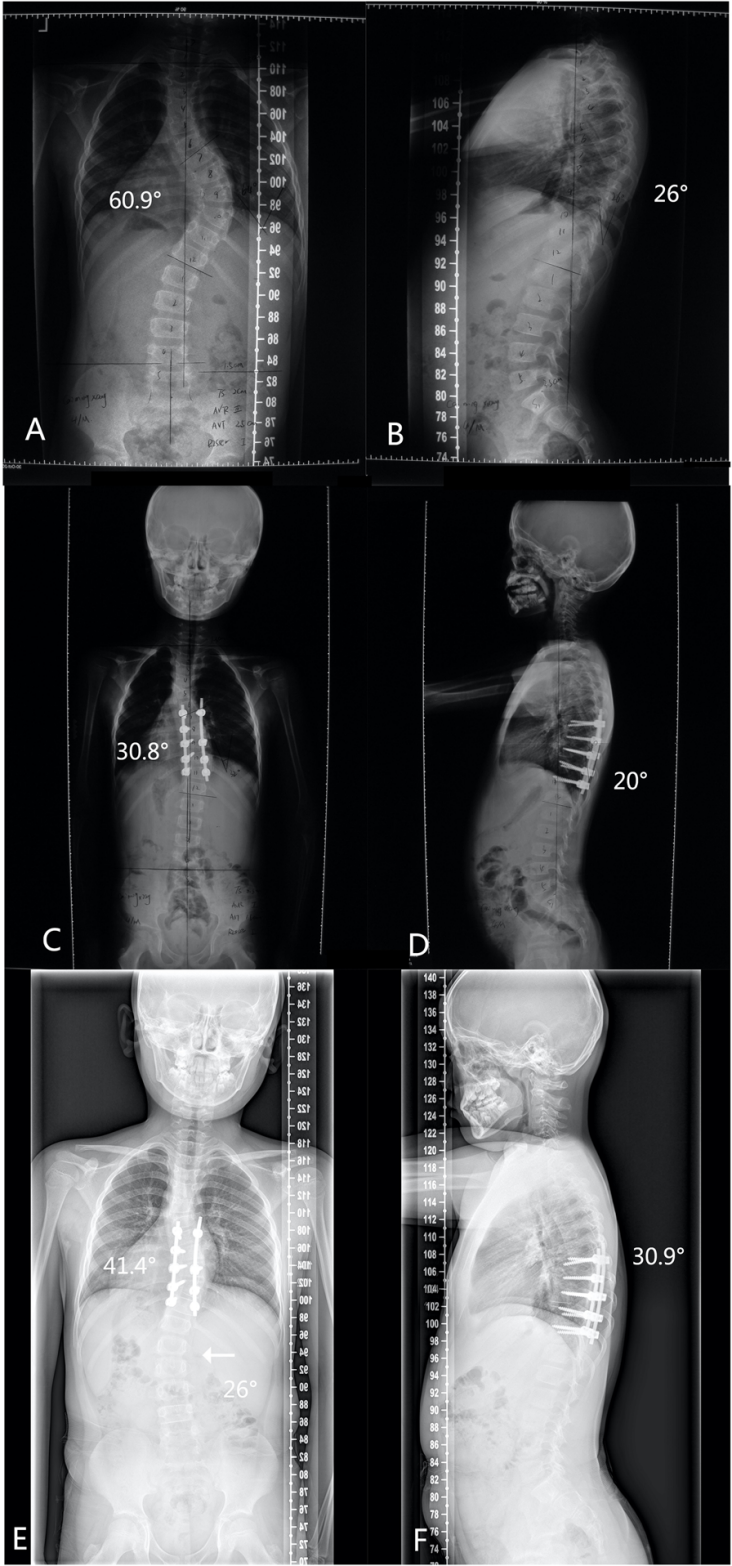
Case 6: Radiographs of a 4-year-old patient with neurofibromatosis and a 60.9°right thoracic scoliosis, who received posterior only fusion operation from T7-T11. A.B. Preoperation. C.D. Postoperation. E.F. The adding on phenomenon (arrow) was obvious at the 34-month follow-up, which was attributed to at the growth of the anterior column of the fusion segments

During the follow-up, the incidence of the alignment complication was relatively high. The fusion block deteriorated more than 10 degrees in 7 patients. Besides the two existing cases, there was a new case of lumbar curve development. With regards to the sagittal alignment, the thoracic-lumbar kyphosis of deteriorated over 20 degrees in 1 case. (Fig. 1).

**Fig. 2:**
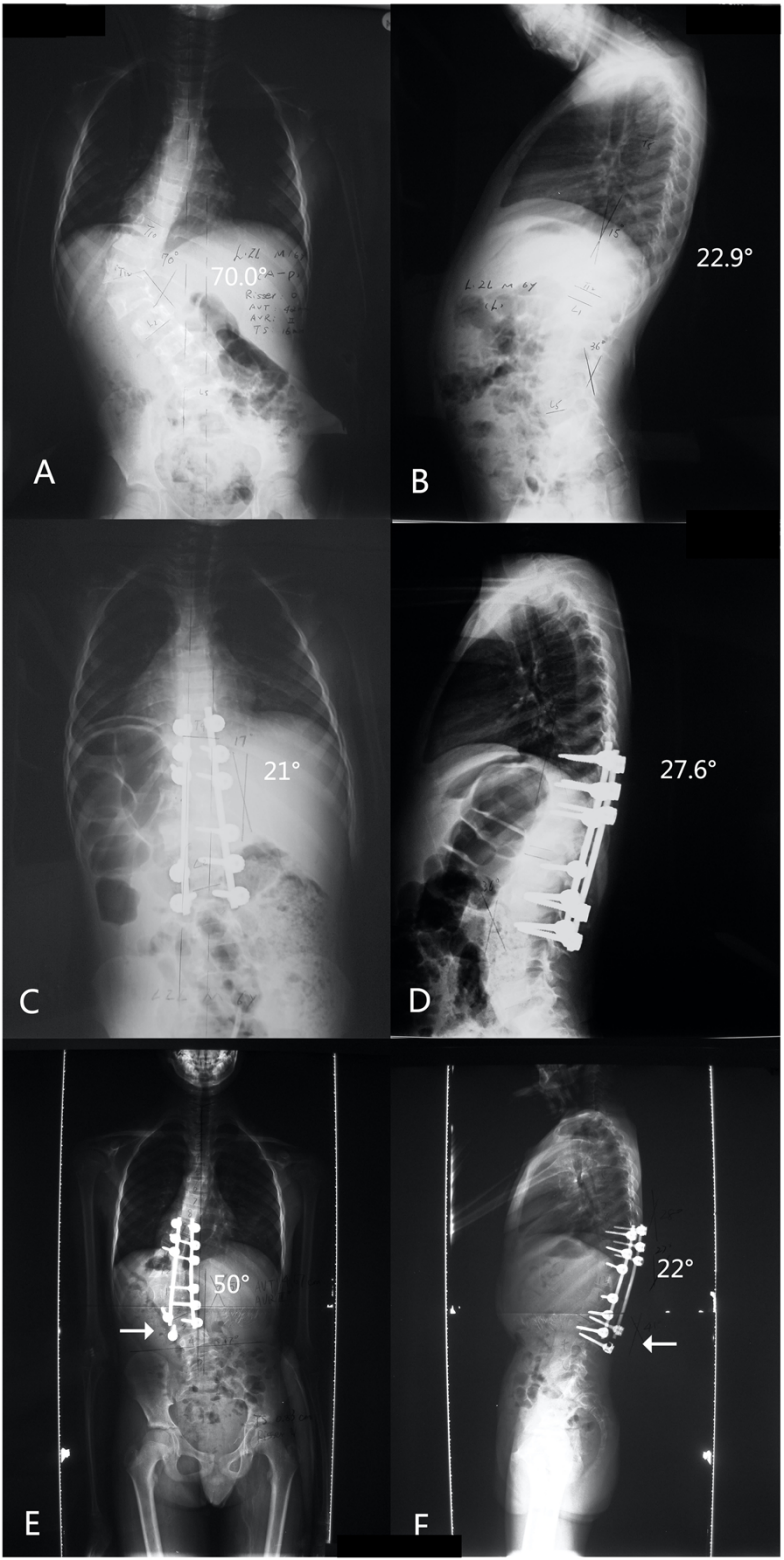
Case 2: A 7-year-old patient with neurofibromatosis and a 70.0°left Thoracolumbar scoliosis, who received posterior only fusion operation from T9-L3. A.B. Preoperation. C.D. Postoperation. E.F. The patient had severe thoracic-lumbar kyphosis deterioration at the 5-year follow-up, and his rod on the convex side slipped from the distal screw track (arrow). The posterior fusion alone was insufficient to inhibit the growth of the fusion segments even using the pedicle screws

However, only 2 patients who suffered alignment problems underwent revision surgeries. In one case, the patient had the severe thoracic-lumbar kyphosis deterioration, and the rod in convex side slipped from the distal screw track. A revision surgery was performed to put the rod back to the screw track, replace the cap, add a trans-connector and augmnt the distal fusion area with allograft. (Fig. 2). In the other case, the two proximal anchor screws of the convex side were pulled out, the instrument of this side was taken off, and the fusion range was extended proximally.

Except for the two cases mentioned above, another patient had one proximal screw dislodged radiographically. As there was no symptom, the revision operation was not taken.

There were no neurological complications (transient or permanent neurological deficiencies) observed.

### Pulmonary functions

The FVC% changed from 91% ± 16% from preoperatively to 95% ± 11% at the last follow-up without statistical difference (5.6% ± 8.6% difference, *P* = 0.57). The forced expiratory volume in one second (FEV1) was not significantly improved at the last follow-up (93% ± 13% vs 97% ± 9%, 5.6 ± 8.6% difference *P* = 0.405) (Table 3).

**Table 3:** Summary of clinical data, body height and pulmonary function on 10 NF-1 patients with early-onset scoliosis treated by posterior only fusion or traditional growing rods

No	Age	FU (Mon)	Pre-Height (cm)	FU-Height (cm)	Pre-O T1S1(cm)	FU T1S1 (cm)	Pre-O T1–12(cm)	FU T1–12(cm)	Pre-% FVC (Present/ Predicted)	FU-% FVC (Present/ Predicted)	Pre-% FEV1 (Present/ Predicted)	FU-% FEV1 (Present/ Predicted)	Pre-ASIA score	FU-ASIA score
1	9.1	24	126	149	33.6	37.9	22.1	25.4	85.4	91.2	106.3	105.0	112	112
2	7.2	61	110	136.5	30.2	34.1	19.6	22.1	87.5	90.0	104.2	91.4	112	112
3	8.3	88	120	148.2	36.1	43.2	22.1	25.6	98.9	99.1	103.1	104.2	112	112
4	4.2	35	85	105.0	38.7	43.9	24.9	27.8	77.0	90.2	81.2	92.5	108	110
5	7.1	49	118	147.1	36.0	41.9	20.4	24.4	90.6	91.6	82.0	86.4	112	112
6	4.1	34	120	140.5	26.7	31.4	16.7	19.7	121.2	120.4	108.3	107.5	112	112
7	9.2	67	131	152.6	42.5	46.7	26.8	29.6	88.4	90.4	87.4	92.1	112	112
8	9.5	68	147	166.9	30.8	36.4	16.7	20.6	62.0	77.2	69.0	76.4	107	107
9	9.6	62	134	163.0	32.4	39.4	20.5	24.0	106.9	104.1	98.3	99.1	112	112
10	9.6	52	142	167.4	38.0	43.2	24.0	25.6	94.4	98.7	90.2	93.1	112	112

*FU* Follow-up, *Pre-O* Preoperative, P*ost-O* Postoperative

## Discussion

In the present study, we evaluated the mid-long term (average 4.5 years follow-up) clinical outcome of posterior only instrumented fusion surgery for NF-1 patients with dystrophic EOS. The major curve was corrected from 66.1° ± 16.2° (43° to 90.3 °), preoperatively, to 31.1 ° ±14.6 ° (13.4 ° to 51.2 °) post-operatively (*P* = 0.00). However, the major curve at the last follow-up fell back to 41.0 ° ±16.0 ° (17 ° to 70.3 °). The T1-S1 length increased by 2.8 cm ±1.0 cm after surgery and increased at a speed of 0.6 cm ±0.3 cm per year. However, the incidence of the alignment complications was relatively high during the follow-up.

About 10% of children with NF-1 develop scoliosis that predominantly involves the cervical and thoracic spine [13]. Dystrophic scoliosis with NF-1 has a high risk of rapid progression [5]. The progression of the dystrophic curve can be neither stopped nor relieved by corset therapy [14]. Therefore, aggressive surgical treatments of dystrophic scoliosis in NF-1 patients was widely recommended [3, 15].

Traditionally, the combined anterior and posterior fusion was supported by majority of people and was recognized as the most reliable method [16–18]. The clinical outcome of dystrophic scoliosis patients treated with posterior-only fusion by using hooks and rods demonstrated that the pseudarthrosis rate was high and curve progression was common [17]. Recently, some good results of posterior only fusion surgeries in NF-1 patients with dystrophic scoliosis have been reported [9, 10, 19]. In this study, we reported our results about the posterior only fusion procedure in NF-1 patients with EOS.

In addition to our study, there are currently only two articles specifically described the outcomes of fusion procedures to treat EOS in NF-1 patients. Greggi et al. [11] reported that NF-1 EOS patients either underwent posterior fusion if the thoracic kyphosis was less than 50°, or underwent anterior-posterior spinal fusion surgery if the thoracic kyphosis was 50° or more. In their study, the average correction rate was 60%, slightly higher than our results. And the follow-up results were good as well, showing no significant progress. Ryoji Tauchi and his colleagues applied the anterior-posterior fusion techniques to all EOS patients before they aged at 10 and the orthopedic effect was more noticeable. The main curve was corrected from 71.2° to 24.1° (66.2%). After a follow-up averaged on 14 years, the result showed that there was no significant progress in scoliosis. Their correction rate was superior to our study in both groups, which might be related to the better anterior release due to the anterior spinal surgery in addition to the posterior fusion procedure.

In contrast to Greggi’s criteria for grouping patients with kyphotic angles, we used posterior orthopedic fusion procedures for all patients and did not separate the patients by kyphosis degrees or deformity disposal. In our patients with a kyphotic angle greater than 50°, the initial postoperative correction rate was 44.5%, the last follow-up correction rate was 31.1%, and the loss rate was 13.4%. In patients with a kyphotic angle less than 50°, the initial postoperative correction rate was 67.5%, the last follow-up correction rate was 43.8%, and the loss rate was 23.7%. Patients with a kyphotic angle more than 50° had both a lower initial correction rate and a lower last follow-up correction rate than those patients who had a kyphotic angle of 50° or less. But the two groups were similar in terms of subsequent orthopedic maintenance. Compared with the anterior and posterior fusion, simple posterior only orthopedic surgery may not be able to satisfy orthopedic maintenance requirement in NF-1 patients with EOS, while simple posterior orthopedic fusion in patients with larger kyphosis may not even meet the requirements of initial orthopedic surgery.

Five years of clinical follow-up showed that crankshaft occurred in 6 out of 10 patients and the Cobb’s angle of fusion segments increased by 10°. The incidence rate of the crankshaft was also significantly higher in children aged at 7 or younger than that of patients who were 7 to 10 years old. Using a single posterior approach orthopedic fusion surgery was at very high risk of the crankshaft, which was consistent with the previous non-surgical observation of such patients [2].

In addition, it has been found that higher density of pedicle screw placement can help to improve the postoperative correction rate of EOS in NF-1patients [10, 19]. We can only perform regular fluoroscopy due to the lack of O-arm at the early stage of our study, which made it difficult to place the pedicle screws. The average ratio of fixed/surgical segments was 0.69. However, we found that there were three patients whose fixed/surgical segment ratio was above the average, reaching 64% on average. The correction rate at the last follow-up was 49%, and the rate of loss was 15% for these three patients. For the other patients, the average postoperative correction rate was 49%, the last follow-up correction rate was 35%, and the correction loss rate was 15%. It is possible that the insufficiency of placed screws on the spine led to the insufficiency of the correction force, which resulted in the poor initial orthopedic effect in our study. However, lacking of enough screws did not significantly affect the result of the surgery in terms of the prevention of scoliosis progress.

It is generally accepted that scoliosis caused by NF-1 is relatively stiff and the preoperative traction may contribute to the improvement of orthopedic effect. However, no specific study has been published to address this specific issue. For most patients with severe scoliosis, preoperative traction can improve the orthopedic effect, but it also increases the medical costs and the distress of patients. And poor compliance was found in patients with EOS due to their young age. Therefore, further evidence is needed to determine the effect of preoperative traction for EOS in NF-1patients.

The average number of fusion segments we performed was 8.1, while it was 13.1 in Tauchi’s study [20]. It suggested that fewer fusion segments in our study led to the poor orthopedic maintenance. In our study, the majority of spine fusion ranges were from upper end vertebrae to lower stable vertebrae, which was adequate for patients with AIS and NF-1 patients with non-dystrophic scoliosis, but insufficient for NF-1 patients with dystrophic EOS. For EOS patients, height retention is one of the factors we must take into account, because maintaining spinal length is a critical factor in allowing adequate lung development, which is a major goal of EOS treatment. The average length of T1-S1 in our patients was 34.5 cm preoperatively, and 39.8 cm at the last follow-up. There was a 5.3 cm growth in length. The length of T1-T12 was 21.4 cm preoperatively and 24.5 cm at the last follow-up, resulting in a 3.1 cm growth. In Tauchi’s study, the average preoperative and last follow-up of T1-S1 were 30.7 cm and 36.2 cm respectively, resulting in a 5.5 cm growth. The preoperative and last follow-up of T1-T12 length was 18.8 cm and 21.9 cm, respectively, resulting in a growth of 3.1 cm. Compared to Tauchi’s study, our surgery was not superior in preserving trunk height. This might be due to the shorter follow-up time, 4.5 years in our study and 14 years in Tauchi’s study, and the fact that patients in our study were young and still have the potential of torso growth.

In this study, there was no significant improvement of lung function brought by the treatment, probably because the patient’s preoperative lung function impairment was not significantly associated with the EOS. On the other hand, the early fusion of the spine did not cause damage to the patient’s lung function, which may be due to the shorter fusion segment we chose.

There was no neurological complication (transient or permanent neurological deficiency) observed. Given the facts that the complication rate of anterior-posterior approach is as high as 64%, the probability of perioperative pulmonary dysplasia is as high as 45%, and the fact that lung injury and dural tear are also seen during the precedure [19, 20], we suggest that posterior fusion surgery be a good alternative way to get a better postoperative outcome and reduce the postoperative complications.

Until now, none of the current approaches to treat EOS in NF-1 patients has reached a balance between maintaining good orthopedic outcomes and reducing complications and patient distress. Recent studies indicated that non-fusion technology is promising. Jain et al. [21] used the growth-bar technique in 14 NF-1 patients with EOS in 5 centers and performed an average follow-up of 54 months. It was shown that the correction rate of the non-fusion technology at the last follow-up was 51% and the annual spine length increase was 1.1 cm. Considering the correction rate and retaining of the space to grow, it is undoubtedly worth the wait for the longer time.

Of course, there are disadvantages of growth rod technology such as the high incidence of complications associated with internal fixation. Given that further validation of non-fusion technology to treat NF-1 patients with EOS is needed, it is undoubtable that posterior only fusion technology is a promising choice to treat the disease.

## Conclusions

We retrospectively reviewed the general clinical data and surgery related data of 10 NF-1 patients with EOS who had undergone posterior only orthopedic internal fixation fusion surgery at Peking Union Medical College Hospital and had an average follow-up duration of 4.5 years. It was found that the orthopedic effect of posterior only fusion method was not good way to correct the scoliosis, especially not good for the maintenance of orthopedic effect. For the surgical treatment of NF-1patients with EOS, the ideal goal of treatments should be to maintain the patients’ height as much as possible while achieving and maintaining a good orthosis in patients, and reducing the risk of surgery and complications at the same time.

## Data Availability

The data that support the findings of this study included in this manuscript, and the original files are available from the corresponding author upon reasonable request.

## Abbreviations

CP: crankshaft phenomenon
EOS: Early-Onset Scoliosis
FEV1: The forced expiratory volume in one second
FU: Follow-up
GR: traditional growing rod
MC: Major curve
NF-1: Neurofibromatosis type 1
PF: posterior only fusion
PI: Post the Initial surgery
Post-O: postoperative
Pre-O: Preoperative

## References

[1] Jett K, Friedman JM (2010). Clinical and genetic aspects of neurofibromatosis 1. Genet Med.

[2] Akbarnia BA, Gabriel KR, Beckman E, Chalk D (1992). Prevalence of scoliosis in neurofibromatosis. Spine (Phila Pa 1976).

[3] Calvert PT, Edgar MA, PJ W. (1989). Scoliosis in neurofibromatosis. The natural history with and without operation. J Bone Joint Surg Br.

[4] Betz R. R. Scoliosis surgery in neurofibromatosis. Clin Orthop Relat Res. 1989;(245):53–6. 2502352

[5] Durrani AA, Crawford AH, Chouhdry SN, Saifuddin A, Morley TR (2000). Modulation of spinal deformities in patients with neurofibromatosis type 1. Spine (Phila Pa 1976).

[6] Flynn JM, Tomlinson LA, Pawelek J, Thompson GH, McCarthy R, Akbarnia BA (2013). Growing-rod graduates: lessons learned from ninety-nine patients who completed lengthening. J Bone Joint Surg Am.

[7] Halmai V, Doman I, de Jonge T, Illes T (2002). Surgical treatment of spinal deformities associated with neurofibromatosis type 1. Report of 12 cases. J Neurosurg.

[8] Tsirikos AI, Saifuddin A, Noordeen MH (2005). Spinal deformity in neurofibromatosis type-1: diagnosis and treatment. Eur Spine J.

[9] Deng A, Zhang HQ, Tang MX, Liu SH, Wang YX, Gao QL (2017). Posterior-only surgical correction of dystrophic scoliosis in 31 patients with neurofibromatosis type 1 using the multiple anchor point method. J Neurosurg Pediatr..

[10] Li Y, Yuan X, Sha S, Liu Z, Zhu W, Qiu Y (2017). Effect of higher implant density on curve correction in dystrophic thoracic scoliosis secondary to neurofibromatosis type 1. J Neurosurg Pediatr.

[11] Greggi T, Martikos K (2012). Surgical treatment of early onset scoliosis in neurofibromatosis. Stud Health Technol Inform.

[12] Ferner RE (2010). The neurofibromatoses. Pract Neurol.

[13] Lykissas MG, Schorry EK, Crawford AH, Gaines S, Rieley M, Jain VV (2013). Does the presence of dystrophic features in patients with type 1 neurofibromatosis and spinal deformities increase the risk of surgery?. Spine (Phila Pa 1976).

[14] Shen JX, Qiu GX, Wang YP, Zhao Y, Ye QB, Wu ZK. Surgical treatment of scoliosis caused by neurofibromatosis type 1. Chinese medical sciences journal = Chung-kuo i hsueh k'o hsueh tsa chih. 2005;20(2):88–92. 16075744

[15] Betz RR, Iorio R, Lombardi AV, Clancy M, Steel HH (1989). Scoliosis surgery in neurofibromatosis. Clin Orthop Relat Res.

[16] Crawford AH (1989). Pitfalls of spinal deformities associated with neurofibromatosis in children. Clin Orthop Relat Res.

[17] Sirois JL, Drennan JC (1990). Dystrophic spinal deformity in neurofibromatosis. J Pediatr Orthop.

[18] Winter RB, Moe JH, Bradford DS, Lonstein JE, Pedras CV, Weber AH (1979). Spine deformity in neurofibromatosis. A review of one hundred and two patients. J Bone Joint Surg Am.

[19] Saygin C, Wiechert A, Rao VS, Alluri R, Connor E, Thiagarajan PS, et al. CD55 regulates self-renewal and cisplatin resistance in endometrioid tumors. J Exp Med. 2017. 10.1084/jem.20170438 PMC5584126 28838952

[20] Tauchi R, Kawakami N, Castro MA, Ohara T, Saito T, Morishita K, et al. Long-term surgical outcomes after early definitive spinal fusion for early-onset scoliosis with Neurofibromatosis type 1 at mean follow-up of 14 years. J Pediatr Orthop. 2017;1. 10.1097/BPO.0000000000001090 31815861

[21] Jain VV, Berry CA, Crawford AH, Emans JB, Sponseller PD (2017). Growing rods are an effective Fusionless method of controlling early-onset scoliosis associated with Neurofibromatosis type 1 (NF1). J Pediatr Orthop.

